# Cancer Stem Cells: What Do We Know about Them?

**DOI:** 10.3390/cells10061528

**Published:** 2021-06-17

**Authors:** Ira Skvortsova

**Affiliations:** 1 Laboratory for Experimental and Translational Research on Radiation Oncology (EXTRO-Lab), Department of Therapeutic Radiology and Oncology, Innsbruck Medical University, A-6020 Innsbruck, Austria; Ira.Skvortsova@i-med.ac.at; Tel.: +43-512-504-27758; Fax: +43-512-504-27756; 2 Tyrolean Cancer Research Institute, A-6020 Innsbruck, Austria

During past decades, survival rates in cancer patients have drastically improved due to the successful development of novel, promising chemical compounds and therapeutic schedules [1]. Currently, cancer researchers are more focused on personalized medicine. Therefore, comprehensive elucidation of the molecular mechanisms that make tumors more aggressive and insensitive to traditional anti-cancer treatment is needed. 

It is known that malignant tumors comprise the non-cancerous cells and heterogeneous subpopulations of cancer cells. Therapeutic approaches can only be curative in the situation where all carcinoma cells are destroyed and eliminated during anti-tumor therapy. Unfortunately, there is a cancer cell subpopulation that is characterized by its abilities to self-renew and to survive after cytotoxic treatment [2,3,4]. These cells are called cancer stem cells (CSCs) or tumor-initiating cells (TICs). Although the CSC hypothesis is debated by cancer researchers, it still receives ongoing, increasing interest in the scientific community. Thus, molecular biologists, pharmacologists, chemists, biochemists, bioinformaticians, clinical researchers put much effort into investigating the physiology and molecular properties of CSCs.

This article collection highlights the role of CSCs in the development and progression of malignant tumors. Thus, Biserova et al. [5] have discussed how glioblastoma cells with stemness properties can contribute to tumor formation, growth and expansion. Stemness properties of glioblastoma cells can be regulated by a number of factors contributing to cell resistance to therapy and recurrence. The authors describe in detail the role of glioblastoma stem cells in the treatment insensitivity of brain tumors. The importance of the development of biomarkers predicting reduced therapy responses is also argued. Additionally, the problem of therapeutic target discovery is reviewed in the context of glioblastoma research.

Another article related to the development of targeted therapeutics for glioblastoma is included in this article collection. Juric et al. [6] have elucidated an important role of transcriptional CDK inhibitors, CYC065 and THZ1, to prevent glioblastoma relapses. They clearly show that transcriptional CDK inhibitors can effectively kill glioblastoma cells with stemness characteristics. These chemical compounds revealed not only cytostatic but also cytotoxic effects (apoptosis) on glioblastoma stem cells.

CSC heterogeneity issues have raised the question of whether molecular patterns of hepatocellular CSCs are equal in two cohorts of patients in Asia and Europe [7]. Although it is possible to assume that the investigated CSCs could differ in their molecular profiling, this comparative study demonstrated that mRNA expression of hepatocellular CSC marker CD90/Thy-1 was comparable in Eastern and Western patient groups. This fact can be successfully utilized in the planning of clinical trials devoted to the diagnostic and therapeutic issues in different populations of patients. Karagonlar et al. [8] enrich the information about the molecular features of hepatocellular CSCs. Thus, the authors have provided experimental confirmation of the role of KLF4 in CSC plasticity and dedifferentiation. This is an important study, showing a novel function of KLF in the modulation of expression of hepatocellular CSC markers.

Dionisio et al. [9] continue to consider CSC markers as predicting factors for unfavorable prognosis in cancer patients. They have reported that breast CSC markers CD44, CD49f, P-cadherin, EpCAM, and ALDH1 can be associated with a higher risk for brain metastasis development and poor clinical outcomes in breast cancer patients. This clinically relevant paper can help clinicians to better stratify their patients for personalized therapeutic management. CSC plasticity makes CSC research more complicated and difficult due to the unstable molecular and pathological properties of CSCs. Although stemness characteristics of triple-negative breast carcinoma (TNBC) cells can be unaffected, cellular metabolism is markedly changed under continuous stimulation of breast carcinoma cells by pro-inflammatory cytokines TNFα and IL-1β [10]. Additionally, pro-inflammatory cytokines are able to promote pro-metastatic cascades in TNBC and reduce the recruitment of monocytes and neutrophils in vivo. Therefore, chronic inflammation can be implicated in the formation of TNBC with more aggressive phenotypes, and this fact should be taken into account by clinicians.

The Guest Editor and authors hope that this Special Issue of *Cells* will be of great interest to scholars involved in cancer research.
